# Down-regulation of transforming growth factor beta-2 expression is associated with the reduction of cyclosporin induced gingival overgrowth in rats treated with roxithromycin: an experimental study

**DOI:** 10.1186/1472-6831-9-33

**Published:** 2009-12-08

**Authors:** Simone Aparecida Probst Condé, Marcus Gomes Bastos, Beatriz Julião Vieira, Fernando Monteiro Aarestrup

**Affiliations:** 1 Post-graduate Program in Health, Federal University of Juiz de Fora/UFJF - Juiz de Fora/Brazil, Dom André Arcoverde Foundation, Department of Clinical Dental - Valença/Brazil; 2 Department of Nephrology, NIEPEN Institute, IMEPEN Foundation, Federal University of Juiz de Fora/UFJF- Juiz de Fora/Brazil; 3 Center of Biology Reprodution, Laboratory of Immunopathology and Experimental Pathology, Federal University of Juiz de Fora - Juiz de Fora/Brazil; 4 Center of Biology Reprodution, Laboratory of Immunopathology and Experimental Pathology, Federal University of Juiz de Fora - UFJF, Suprema Instituition, Faculty of Medicine, Department of Semiology - Juiz de For a/Brazil

## Abstract

**Background:**

Gingival overgrowth (GO) is a common side effect of the chronic use of cyclosporine (CsA), an immunosuppressant widely used to prevent rejection in transplant patients. Recent studies have reported elevated levels of specific cytokines in gingival overgrowth tissue, particularly TGF-beta, suggesting that this growth factor plays a role in the accumulation of extracellular matrix materials. The effectiveness of azithromycin, a macrolide antibiotic, in the regression of this undesirable side effect has also been demonstrated.

**Methods:**

In this study, we created an experimental model for assessing the therapeutic effect of roxithromycin in GO and the expression of transforming growth factor beta (TGF-beta2) through immunohistochemistry. We used four groups of rats totaling 32 individuals. GO was induced during five weeks and drug treatment was given on the 6th week as follows: group 1 received saline; group 2 received CsA and was treated with saline on the 6th week; group 3 received CsA and, on the 6th week, ampicilin; and group 4 received CsA during 5 weeks and, on the 6th week, was treated with roxithromycin.

**Results:**

The results demonstrated that roxithromycin treatment was effective in reducing cyclosporine-induced GO in rats. Both epithelial and connective tissue showed a decrease in thickness and a significant reduction in TGF-beta2 expression, with a lower number of fibroblasts, reduction in fibrotic areas and decrease in inflammatory infiltrate.

**Conclusion:**

The present data suggest that the down-regulation of TGF-beta2 expression may be an important mechanism of action by which roxithromycin inhibits GO.

## Background

Gingival overgrowth (GO) is a common side effect of certain drugs, such as phenytoin, calcium channel blockers and cyclosporin [1,2].

Cyclosporine (CsA) has been widely used to prevent organ transplant rejection and to treat various immunodiseases [2]. It selectively suppresses helper T-cell function and modulates the network of inflammatory cytokines. However, CsA is associated with several adverse effects, such as nephrotoxicity, hepatotoxicity, hirsutism and gingival overgrowth (GO) [1,3,4]. The average prevalence of dentate transplant patients who develop cyclosporine induced GO is around 30%, with variation between 10 and 85% [5-9]. When associated with other medication, such as calcium channel blocker antihypertensives, this prevalence increases, as does the gravity of the complication, and, consequently, the risk [10-13].

Fibrosis, one of the most important finding in GO, is the result of a variety of biochemical signals from many cell types including inflammatory cells and fibroblasts, which stimulate fibroblast proliferation and extracellular matrix production [14]. The transforming growth factor beta (TGF-beta) is a multifunctional family of cytokines present in this pathway and there are three mammalian isoforms of TGF-beta (TGF- beta1, TGF-beta2 and TGF-beta3), which are structurally similar [15]. Increased TGF-beta1 levels have been associated in cyclosporin-induced renal fibrosis [16]. Most of the studies argue in favor of TGF-beta1 in the CsA-induced gingival overgrowth. However a few number of studies investigated the expression of TGF-beta2 in this pathological condition [15,17]. TGF-beta2 is considered an immunosuppressive cytokine modulated by cyclosporine that plays a central role during the formation of fibrosis and inflammatory reaction [18-20]. Recently, it was demonstrated that roxithromycin has an inhibitory effect on the production of TGF-βbeta by human mesangial cells, and may be efficient in the treatment of glomerulosclerosis [21]. Therefore, this macrolide antibiotic with similar characteristics of azithromycin, is known to have anti-inflammatory, immunomodulatory and tissue reparative effects besides its bacteriostatic activity may be a therapeutic alternative in the treatment of gingival overgrowth. Recently, we published a study with interesting results in renal transplanted patients in which the gingival overgrowth was reduced after roxithromycin treatment [22]. In the present study we investigated the effect of roxithromycin on GO in rats treated with cyclosporine. Our findings indicate that roxithromycin reduces GO and down-regulates TGF-beta2 expression in gingival tissues.

## Methods

Thirty-two 6-week-old male Wistar rats (*Rattus novergicus albinun*), weighing 100 to 150 g. were randomly selected and divided equally into four groups of eight animals each. The control rats (group 1) were daily injected subcutaneously with saline. The experimental rats (group 2) were treated with CsA injected subcutaneously in a daily dose of 10 mg/kg for six weeks and, at the beginning of the 6th week, they were injected subcutaneously with saline. Group 3 was similar to group 2, but in the last week it was treated with 50 mg/kg ampicillin (an antibiotic widely used in periodontal infections) via gastric feeding. In group 4, the animals received CsA daily for 6 weeks and in the last week they were treated with 40 mg/kg roxithromycin by gastric feeding. The rats were weighed weekly and the dosage was adjusted accordingly. Diet and drinking water were given *ad libitum *during the experiment.

### Macroscopy analysis

At the end of the experimental period, all the animals were sacrificed by means of an overdose of anesthesia (Kensol^® ^- 10 mg/kg and Vetanarcol^® ^- 90 mg/kg). To investigate gingival alterations over time, stone models were made from silicone impressions of the maxillary anterior region. The higher buccal-lingual width and mesio-distal width of the anterior segment of the maxilla were measured using a digital caliper.

### Histopathology and histomorphometry

All gingival samples obtained were fixed in 10% buffered formalin (pH 7) for at least 24 hours. After fixation, the samples were gradually dehydrated in increasing concentrations of ethanol (70% to 100%), cleared in xylene, soaked and embedded in paraffin, according to routine histological methods.

The paraffin-embedded fragments were cut with an "820" Spence microtome and 4 μm thick sections were obtained. All gingival samples were included in paraffin with the same orientation. The sections analysed always showed the epithelium, the connective and muscular tissue. The histometry measurements were performed since the ephitelium until the muscular tissue. The histological slides were kept in an incubator to dry, and then the sections were stained with hematoxylin and eosin for histological analysis. Histomorphometry was performed using images captured and evaluated by a computerized Axion Vision (Zeiss, Berlin, Germany) image capture system. Images were captured from four randomly chosen microscopic fields for each histological slide, using the digital camera (400× and 100× magnification) of an Axiostar Plus microscope (Zeiss, Berlin, Germany) and the number of TGF-betapositive cells were counted.

The images were stored and submitted to a count of inflammatory cells and of all fibroblast-like cells. Linear measurements were made to obtain the higher diameter of epithelial and gingival connective tissue, using digital marking.

### Immunohistochemistry

Paraffin sections (4 μm of thick) of formalin-fixed tissue were obtained at varying depths from each tissue block. The presence of TGF-beta2 protein in the gingival samples was investigated in paraffin sections by using the avidin-biotin-peroxidase complex procedure (ABC) with an additional step for antigen retrieval with a retrieval solution (Dakopatts, Copenhagen, Denmark). In brief, the sections were incubated with polyclonal rabbit anti-TGF-beta2 antibody diluted 1/20 and incubated for one hour at room temperature (Santa Cruz, Santa Cruz, USA) followed by a goat anti-rabbit IgG biotinylated antibody (Dakopatts, Copenhagen, Denmark). Controls for the ABC procedure were performed by replacing anti-TGF-beta2 antibody with normal rabbit serum, or by omitting the anti-TGF-beta2 antibody. The sections were analyzed via light microscopy, and photomicrographs were taken with an Axiostar Zeiss microscope (Zeiss, Germany). The number of the TGF-beta2 immunoreactive cells by microscopic field (400 × magnification) was counted in connective tissue.

### Statistical analysis

Data were expressed as means and standard deviation. ANOVA was used for statistical evaluation and the Bonferroni post-hoc test was used after ANOVA. The gingival mucosa width, the number of inflammatory cells, all fibroblast-like cells and TGF-beta2 immunoreactive cells were compared between groups by using the Student's *t*-test. The significance level adopted was *p *< 0.05.

The manuscript has been performed with the approval of ethics committee in experimental research on animals by Federal University of Juiz de Fora/Brazil (protocol n° 039/2005).

## Results

**Figure 1:**
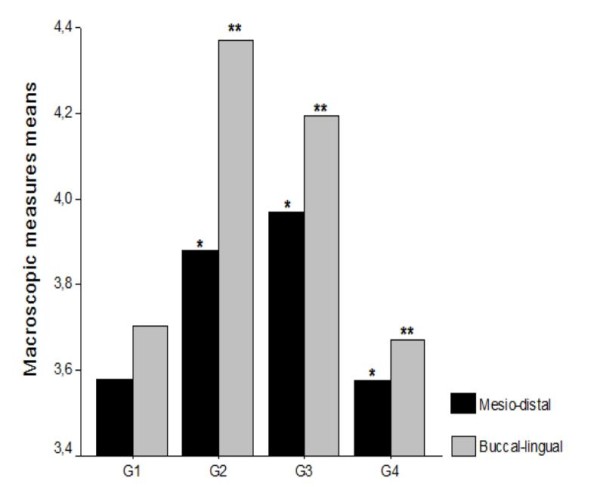
**Macroscopic measurements of buccal-lingual and mesio-distal maxillary regions**. Results expressed as mean and the level of significance for statistical analyse was p < 0.05. G1 (n = 8 animals) - negative control group - administration of saline solution. G2 (n = 8 animals) - positive control group - administration of CsA + saline. G3 (n = 8 animals) - positive control group- - administration of CsA + AMP. G4 (n = 8 animals) - treated group - administration of CsA + ROX.

**Figure 2:**
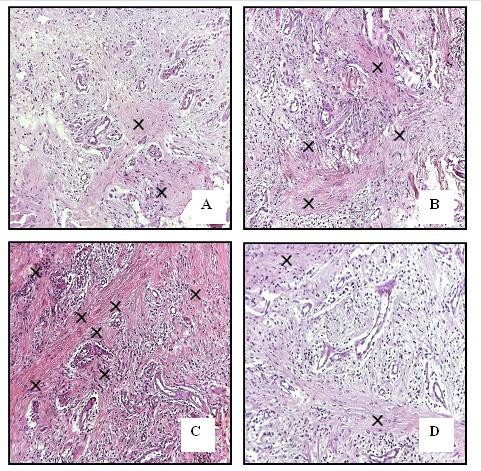
**Representative microscopic fields**. A: Normal connective tissue in group 1 (negative control); B-C: Extensive fibrosis in groups 2 and 3 (positive controls); D: Few areas with fibrosis in group 4 (treated group). Original magnificance 400×.

**Figure 3:**
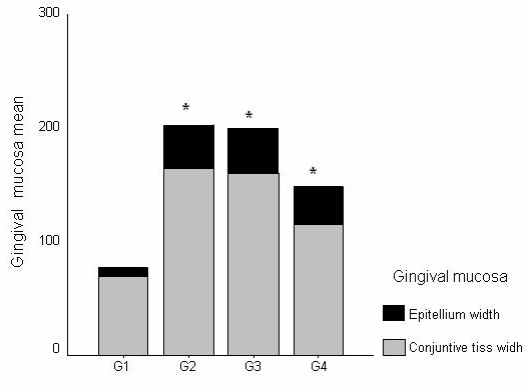
**Measurements of gingival mucosa (epithelium and lamina propria)**. Results expressed as mean (p < 0.05). G1 (n = 8 animals) - negative control group - administration of saline solution. G2 (n = 8 animals) - positive control group - administration of CsA + saline. G3 (n = 8 animals) - positive control group- - administration of CsA + AMP. G4 (n = 8 animals) - treated group - administration of CsA + ROX.

**Figure 4:**
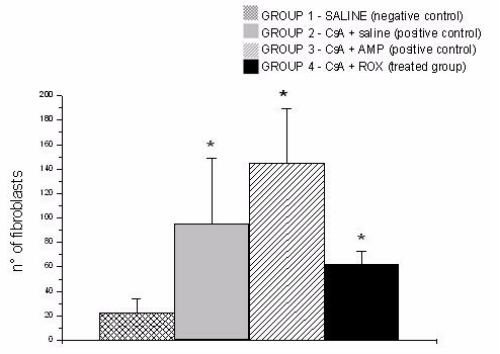
**Numbers of fibroblasts per microscopic fields**. Five microscopic fields with 400× magnification were studied per histological slides. Results expressed as mean (p < 0.05). G1 (n = 8 animals) - negative control group - administration of saline solution. G2 (n = 8 animals) - positive control group - administration of CsA + saline. G3 (n = 8 animals) - positive control group- - administration of CsA + AMP. G4 (n = 8 animals) - treated group - administration of CsA + ROX.

**Figure 5:**
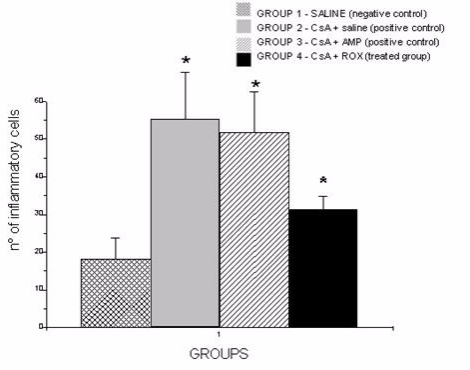
**Number of inflammatory cells per microscopic fields**. Five microscopic fields with 400× magnification were studied per histological slides. Results expressed as mean ± SD (p < 0.05). G1 (n = 8 animals) - negative control group - administration of saline solution. G2 (n = 8 animals) - positive control group - administration of CsA + saline. G3 (n = 8 animals) - positive control group- - administration of CsA + AMP. G4 (n = 8 animals) - treated group - administration of CsA + ROX

**Figure 6:**
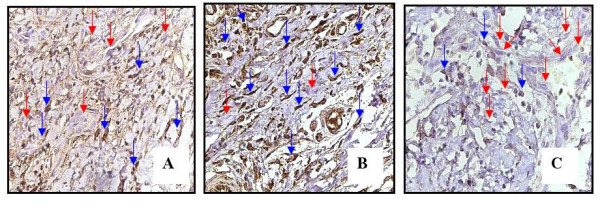
**Representative samples indicated elevated expression of TGF-beta2 in cells of connective tissue (groups 2 and 3); C: Light TGF-beta2 stained cells in group 4**. Red arrows - negative staining cells. Blue arrows: positive staining cells. Original magnificance 400×.

**Figure 7:**
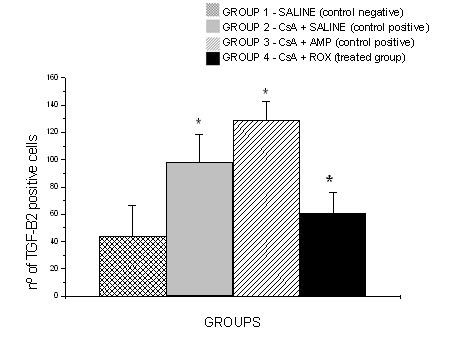
**Number of positive staining cells of TGF-beta2 in connective tissue per microscopic field**. Five microscopic fields with 400× magnification were studied per histological slides. Results expressed as mean mean ± SD (p < 0.05). G1 (n = 8 animals) - negative control group - administration of saline solution. G2 (n = 8 animals) - positive control group - administration of CsA + saline. G3 (n = 8 animals) - positive control group- - administration of CsA + AMP. G4 (n = 8 animals) - treated group - administration of CsA + ROX.

All cyclosporine treated rats presented GO that was evident in all gingival localizations. Graphic 1 shows macroscopic measurements of the buccal-lingual width and mesio-distal width (p < 0.05). The macroscopic analysis revealed a significant reduction of the buccal-lingual width and mesio-distal width in the roxithromycin treated rats (group 4) when compared with group 2 and group 3 (graphic 1) (p < 0.05). The histopathological analysis revealed that GO was a result of the enlargement of epithelial and connective tissues (figures 1A, 1B, 1C, 1D). A moderate acanthosis was observed in animals from groups 2, 3 and 4. No statistical differences were observed when compared the thickness of the epithelium detected in gingival samples obtained from the control positive groups with the treated groups. A severe hyperplasia of collagen fibres were observed in the gingival samples obtained from groups 2 and 3. However, the microscopic study revealed a reduction of collagen fibres in gingival samples of the treated rats when compared with gingival samples of the control rats. No fibrotic areas were observed in gingival samples from group 1 (negative control group). Extensive fibrosis is a common finding in gingival samples from groups 2 and 3, however a few number of fibrotic areas were observed in gingival tissue of roxithromycin treated rats (figure 2). Diameter measurements of the epithelial and connective tissues were obtained (figure 3). The measurements observed in roxithromycin treated rats (group 4) were statistically lower than those observed in the GO positive control groups (group 2 and group 3). The histomorphometric analysis also demonstrated that the animals treated with roxithromycin presented a lower number of fibroblasts per microscopic field (figure 4). The identification of fibroblasts, based on morphological criteria, was made by two different researchers. Finally, a lower number of inflammatory cells was observed in gingival samples obtained from roxithromycin treated group when compared with group 2 and group 3 (figure 5). Cytoplasmatic staining for TGF-beta2 was detected in most cell types of the connective tissue. TGF-beta2 was expressed within connective tissue by inflammatory cells, fibroblasts and endothelial cells (figure 6). Most of the fibroblasts and endothelial cells were TGF-beta2 positive and showed a variable staining pattern, usually weakly or darkly positive (figure 6). Immunohistochemical analysis showed a lower number of TGF-beta2 positive cells (figure 7) - recognized morphologically as fibroblasts, endothelial cells and inflammatory cells in the connective tissue samples from roxithromycin treated rats when compared with GO positive controls rats (group 2, and group 3) (figure 7). No significant differences were detected when compared to the number of TGF-beta2 positive cells in gingival samples obtained from group 1 and group 4 (figure 7).

## Discussion

Cyclosporine (CsA) is a hydrophobic neutral cyclic polypeptide composed of eleven amino acids and made from the fungus *Tolypocladium inflatuns gams*. It specifically acts in suppressing the immune response mediated by T cells. It goes through biotransformation in the liver, resulting in 14 metabolic products, of which 90% is excreted in feces and 10% eliminated by the kidneys [23].

Gingival overgrowth is one of the side effects caused by the chronic use of cyclosporine (CsA), which is employed to prevent rejection of transplanted organs and to treat several autoimmune diseases, such as diabetes mellitus, Behcet's disease, psoriasis, multiple sclerosis, erosive lichen planus, systemic lupus erythematosus, bullous pemphigoid, rheumatoid arthritis, myasthenia gravis, uveitis, and various glomerulopathies [3,24,25].

Several studies have demonstrated elevated levels of specific cytokines in overgrowth gingival tissue, especially TGF-beta, a multifunctional inflammatory mediator, which suggests that this growth factor plays a role in the accumulation of the extracellular matrix [14,25,26], including collagenous proteins [27,28]. The TGF-beta isoforms may be expressed by most cells, including gingival inflammatory cells, endothelial cells and fibroblasts [17]. Elevated gingival TGF-beta1 and TGF-beta2 have been suggested by immunohistochemical study on phenytoin and nifedipine-induced GO [29]. and our data indicate elevated levels of TGF-beta2 expression on cyclosporin-induced GO.

GO treatment includes removing bacterial plaque, maintaining adequate oral hygiene, and also includes invasive procedures, such as gingivectomy. Treatment for this complication, until recently, was only surgical. Nowadays, several studies have been conducted to determine the effect of antibiotic treatment on the regression of gingival overgrowth. In 1995, Wahlstrom, Zamora and Teichmann coincidentally used azithromycin - a semi synthetic antibiotic, derived from the macrolide erythromycin - to treat respiratory infections in two renal transplant patients who had cyclosporine induced GO. They observed gingival reduction after using it. The daily treatment with 500 mg for five days is simple, cheap, conservative, quickly effective, and avoids gingival surgery. It acts mostly against gram-positive and negative bacteria, has quick oral absorption, does not alter serum levels of cyclosporine or levels of creatinine. Azithromycin is well tolerated and associated an efficient oral hygiene program induce a gingival overgrowth reduction [28], but can produce side effects, such as diarrhea, abdominal pain, nausea and vomiting [28-33].

Recently, we used roxithromycin in four renal transplanted patients and the results suggest that roxithromycin may be an important therapeutic tool used to reduce cyclosporine-induced GO [22]. Yamabe et al. (2006) suggested that the use of roxithromycin has an inhibitory effect on the production of TGF-beta by human mesangial cells, and can be efficient in the treatment of glomerulosclerosis. The mechanisms that roxithromycin inhibited TGF-beta production are not clear, but in this study the drug did not inhibit the activation of tyrosine kinase and MAP kinase by thrombin. Roxithromycin suppressed the thrombin-induced translocation of NF-kB p65 protein into the nucleus. It is suggested that the activation of NF-kB regulates TGF-beta production, thus ROX inhibited TGF-beta production via the inhibition of NF-kB.

## Conclusion

In the present study, the results demonstrated that roxithromycin treatment was effective to reduce cyclosporine-induced GO in rats. Both epithelial and connective tissues showed a decrease in thickness in roxithromycin treated rats in comparison with animals from the control groups. In addition, the significant reduction in TGF- beta2 expression in roxithromycin treated rats was associated with a lower number of fibroblasts, with reduction of fibrotic areas and decrease in inflammatory infiltrate. Taken together, our data suggest that the down-regulation of TGF-beta2 expression may be an important mechanism of action by which roxithromycin inhibits GO.

## Abbreviations

AMP: ampicillin; CsA: ciclosporine; GO: gingival overgrowth; MAP quinase: quinase protein actived by mithogen; NF-kB: nuclear factor kappa B; ROX: roxithromycin; TGF-beta1: transforming growth factor beta1; TGF-beta2: transforming growth factor beta2; UFJF: Federal University of Juiz de Fora

## Competing interests

The authors declare that they have no competing interests.

## Authors' contributions

SC have been involved in drafting the manuscript, acquisition and analysis of data and carried out the immunohistochemistry. MB participated in the design of the study and coordination such as performed the statistical analysis. BV analysis and interpretation of data. IM helped in the experimental phase, inducing gingival overgrowth. LV carried out the immunohistochemistry. FA participated in the design of the study and coordination and have given final approval of the version to be published. All authors read and approved the final version of the manuscript.

## Pre-publication history

The pre-publication history for this paper can be accessed here:

http://www.biomedcentral.com/1472-6831/9/33/prepub
